# Carbapenemase-Producing *Klebsiella pneumoniae* From Transplanted Patients in Brazil: Phylogeny, Resistome, Virulome and Mobile Genetic Elements Harboring *bla*_KPC–__2_ or *bla*_NDM–__1_

**DOI:** 10.3389/fmicb.2020.01563

**Published:** 2020-07-15

**Authors:** Otávio Hallal Ferreira Raro, Ravena Maya Cardoso da Silva, Edison Moraes Rodrigues Filho, Teresa Cristina Teixeira Sukiennik, Claudio Stadnik, Cícero Armídio Gomes Dias, Jesús Oteo Iglesias, María Pérez-Vázquez

**Affiliations:** ^1^Departamento de Ciências da Saúde, Universidade Federal de Ciências da Saúde de Porto Alegre – UFCSPA, Porto Alegre, Brazil; ^2^Laboratorio de Referencia e Investigación en Resistencia a Antibióticos e Infecciones Relacionadas con la Asistencia Sanitaria, Centro Nacional de Microbiología, Instituto de Salud Carlos III – CNM-ISCIII, Madrid, Spain; ^3^Departamento de Cuidados Intensivos, Hospital de Clínicas de Porto Alegre – HCPA, Porto Alegre, Brazil; ^4^Serviço de Controle de Infecção, Santa Casa de Misericórdia de Porto Alegre – SCMPA, Porto Alegre, Brazil

**Keywords:** transplanted patients, *bla*_KPC–__2_, *bla*_NDM–__1_, whole-genome sequencing, cgMLST, epidemic clones

## Abstract

**Objectives:**

Carbapenemase-producing *Klebsiella pneumoniae* (CP-Kp) is a major cause of infections in transplanted patients and has been associated with high mortality rates in this group. There is a lack of information about the Brazilian structure population of CP-Kp isolated from transplanted patients. By whole-genome sequencing (WGS), we analyzed phylogeny, resistome, virulome of CP-Kp isolates, and the structure of plasmids encoding *bla*_KPC–__2_ and *bla*_NDM–__1_ genes.

**Methods:**

One *K. pneumoniae* isolated from each selected transplanted patient colonized or infected by CP-Kp over a 16-month period in a hospital complex in Porto Alegre (Brazil) was submitted for WGS. The total number of strains sequenced was 80. The hospital complex in Porto Alegre comprised seven different hospitals. High-resolution SNP typing, core genome multilocus sequence typing (cgMLST), resistance and virulence genes inference, and plasmid reconstruction were performed in 80 CP-Kp.

**Results:**

The mortality rate of CP-Kp colonized or infected transplanted inpatients was 21.3% (17/80). Four CP-Kp epidemic clones were described: ST11/KPC-2, ST16/KPC-2, and ST15/NDM-1, all responsible for interhospital outbreaks; and ST437/KPC-2 affecting a single hospital. The average number of acquired resistance and virulence genes was 9 (range = 2–14) and 27 (range = 6–36), respectively. Two plasmids carrying the *bla*_KPC__–__2_ were constructed and belonged to IncN and IncM types. Additionally, an IncFIB plasmid carrying the *bla*_NDM–__1_ was described.

**Conclusion:**

We detected intrahospital and interhospital spread of mobile structures and international *K*. *pneumoniae* clones as ST11, ST16, and ST15 among transplanted patients, which carry a significant range of acquired resistance and virulence genes and keep spreading across the world.

## Introduction and Objective

Carbapenemase-resistant Enterobacterales (CRE) infection or colonization is a threat to organ transplant recipients (OTRs). The mortality rates in OTR range from 30% to 50% in infections caused by CRE (Satlin et al., 2014; Xu et al., 2017), and when it is focused only in *Klebsiella pneumoniae*, this risk of death increases 10-fold (Brizendine et al., 2015; Lanini et al., 2015), resulting in a worldwide public health emergency, because these microorganisms have been reported in all continents (Nordmann et al., 2011; Satlin et al., 2014). In 2017, the World Health Organization (WHO) released a report (World Health Organization [WHO], 2017b) that marked carbapenem-resistant *K. pneumoniae* (CR-Kp) as a matter of international concern as one of the major causes of hospital-acquired infections. In addition, CR-Kp was also included in the global priority list of antibiotic-resistant bacteria as a critical pathogen by the WHO (World Health Organization [WHO], 2017a).

The most important mechanisms of carbapenem resistance in Enterobacteriaceae are the plasmid-borne carbapenemases. The most common carbapenemases in Enterobacteriaceae are *K. pneumoniae* carbapenemase (KPC; class A); Verona integron-encoded metallo-β-lactamase, imipenemase, and New Delhi metallo-β-lactamase (NDM; class B); and the OXA-48 types (class D). Many carbapenemase genes are carried in different plasmid types (Poirel et al., 2011; Pitout et al., 2015; Raro et al., 2019).

The first study to report the detection of carbapenemases in Brazil was published in 2009 (Monteiro et al., 2009) and described the presence of KPC-2 in *K. pneumoniae* from four patients in the city of Recife, Pernambuco, Brazil, 10 years after the first detection of KPC-2 in the world, in North Carolina, United States (Yigit et al., 2001; Queenan and Bush, 2007). Subsequently, KPC-2 has been described in other species of Enterobacterales distributed throughout the country, but *K. pneumoniae* is the most frequent species carrying carbapenemases. New Delhi metallo-β-lactamase was first detected in 2008 in a Swedish patient who traveled to New Delhi, India (Yong et al., 2009); 5 years later came the first report of an NDM producer strain in Brazil, which was *Providencia rettgeri* isolated from a patient in the city of Porto Alegre, Rio Grande do Sul, Brazil (Carvalho-Assef et al., 2013). After 2009, sporadic cases of carbapenemase-producing Enterobacteriaceae (CPE) were described in Brazil, including the coproduction of NDM-1 and KPC-2 (Pereira et al., 2015; Quiles et al., 2015).

Dissemination and outbreaks caused by KPC- and NDM-producing *K. pneumoniae* isolates have been reported, but we call attention to the scarcity of reports of CPE isolates colonizing or infecting transplanted patients reported so far (Taglietti et al., 2013; Lee et al., 2018).

In the present study, we aimed to describe the phylogeny, resistome, virulome, and the plasmids encoding *bla*_KPC_ and *bla*_NDM_ of CP-Kp strains isolated from transplanted inpatients in a tertiary hospital complex from the city of Porto Alegre, Brazil.

## Materials and Methods

### Clinical Data and Strain Collection

In this study, a total of 80 transplanted inpatients admitted to a 1,000-bed tertiary hospital complex in Porto Alegre, from August 2017 to November 2018 were screened in an active surveillance program to detect carbapenemase-producing *K. pneumoniae*. During this surveillance program, rectal swabs of all patients were collected and screened in admission and once a week for detection of CPE. At the laboratory, a screening procedure was performed using disk diffusion to detect carbapenem resistance. If reduced susceptibility was observed, the isolates were subjected to CarbaNP phenotypic test (Nordmann et al., 2012) following complex hospital’s infection control service and microbiology laboratory recommendations. The hospital complex made up of seven different specialist hospital buildings, including a national referral transplant hospital, and is responsible for providing services to the metropolitan region of Porto Alegre, which comprises more than 4 million people in the south of Brazil (Instituto Brasileiro de Geografia e Estatística [IBGE], 2010). The criteria used to include the patients were as follows: (1) to be a solid organ or marrow bone transplanted patient; (2) to be admitted to the hospital complex during August 2017 and November 2018, and (3) to be colonized by CP-Kp after the transplant surgery (post-operative period). Patient movement networks among the hospitals were characterized through Inkscape v0.92.4 vector graphics editor^1^.

One CP-Kp isolate per patient was collected always in the post-operative period. Infection isolates were prioritized over colonization isolates when both were present. When multiple isolates were detected from different infection sites from the same patient, isolates collected from invasive infections (blood) were always prioritized over isolates from other non-invasive sites. And, when multiple isolates from the same clinical site were present, the first isolate collected was prioritized. The identification of the strains and carbapenemase production was confirmed using MALDI-TOF MS (matrix-assisted laser desorption ionization time of flight; Bruker Daltonics, BD, Bremen, Germany) and CarbaNP phenotypic test (Nordmann et al., 2012) after detection of reduced susceptibility to at least one carbapenem antibiotic by disk diffusion. Disk diffusion tests were then performed for other antimicrobial agents (ampicillin, piperacillin-tazobactam, ampicillin-sulbactam, cefazolin, cefuroxime, cefepime, amikacin, gentamicin, ciprofloxacin, norfloxacin, nitrofurantoin, and sulfamethoxazole-trimethoprim) in all clinical isolates according to the Clinical and Laboratory Standards Institute (CLSI) guidelines (Clinical and Laboratory Standards Institute, 2018). The reference strains used in the disk diffusion technique were *Escherichia coli* ATCC 25922, *Pseudomonas aeruginosa* ATCC 27853 and *K. pneumoniae* ATCC 700603. For CarbaNP assay, *K. pneumoniae* ATCC BAA-1705 and *K. pneumoniae* ATCC BAA-1706 were used as positive and negative controls, respectively, according to the CLSI guidelines (Clinical and Laboratory Standards Institute, 2018). This strategy was performed according to the complex hospital’s infection control service and microbiology laboratory recommendations. Whole-genome sequencing (WGS) was performed in all 80 CP-Kp isolates.

### DNA Extraction, Genomic Library Preparation, and Sequence Analysis

DNA from the isolates was extracted using a QIAamp^®^ DNA mini kit (Qiagen^®^, Hilden, Germany) according to the manufacturer’s instructions. Genomic DNA paired-end libraries were generated using the Nextera XT DNA sample preparation kit (Illumina Inc., San Diego, CA, United States). These libraries were sequenced using the Illumina HiSeq 500 next-generation sequencer with 2 × 150-bp paired-end reads (Illumina Inc). Raw sequence data were submitted to the European Nucleotide Archive (PRJEB34380). The quality of the high-throughput sequence data was assessed by FastQC (Andrews, 2010), and these short reads were subsequently assembled *de novo* into contigs using SPAdes 3.9.0 (Bankevich et al., 2012) testing five different kmers under parameters optimized to give the best assembly, which quality was evaluated by QUAST^2^ (Gurevich et al., 2013). Scaffolding was performed with SSPACE (Boetzer et al., 2011), and GapFiller was used to close sequence gaps (Nadalin et al., 2012). Automatic *de novo* annotation of draft genomes was done using Prokka v1.12-beta (Seemann, 2014).

Illumina sequence reads of *K. pneumoniae* isolates were mapped to the chromosome of *K. pneumoniae* NTUH-K2044 (accession no. NC_012731.1) using Snippy to detect SNPs among all samples^3^. Additionally, Gubbins software was used to eliminate recombinant regions (Croucher et al., 2015). Sequence reads were mapped to an average of 91.97% of the reference genome, with a mean depth of 153x in mapped regions across the isolates. Finally, we generated a concatenated alignment with 52,139 SNP sites. Whole-genome sequencing quality data are detailed in Supplementary Table S1.

### Phylogenetic Analysis

A core genome multilocus sequence typing (cgMLST) that relies on species-specific schemes with a fixed number of chromosomal target genes was applied. For *K. pneumoniae*, we applied a published and public scheme of 2,567 genes (Pérez-Vázquez et al., 2019). This scheme was used to compare *K. pneumoniae* Brazilian isolates with all publicly available complete genomes of *K. pneumoniae* of the same STs. A minimum spanning tree was reconstructed through Ridom SeqSphere + software (Ridom GmbH, Münster, Germany) to analyze the results.

A maximum likelihood phylogenetic tree was reconstructed using SNPs within the core genome using RAxML v7.0.4 (Stamatakis, 2006) with a general time-reversible model and gamma correction for among site rate variation. The SNP alignment of each sequence type was used to recalculate individual maximum likelihood phylogenetic trees. The support for the nodes on the trees was assessed using 100 bootstrap replicates. MEGA X software (Kumar et al., 2018) was used to detect SNPs among all samples.

### Analysis of Antimicrobial Resistance, Virulence Genes, and Plasmid Reconstruction

Antimicrobial resistance genes were analyzed using ResFindertool (CGE server)^3^ with an ID threshold of 98% except for β-lactamase variants, which were determined with a 100% identity. Additionally, SRST2 (Inouye et al., 2014) was used to detect resistance genes and alleles with the ARGannot database (Gupta et al., 2014). Virulence genes were identified using the BIGSdb-Kp database (Institut Pasteur, last accessed March 2019)^4^ (Bialek-Davenet et al., 2014). Capsule K-locus and LPS O-antigen typing were characterized using WGS data through Kaptive tool (Wyres et al., 2016; Wick et al., 2018)^5^. To reconstruct the plasmids of each genome, an in-house script was used^6^ as described (Pérez-Vázquez et al., 2019).

## Results and Discussion

### Clinical and Demographic Data

The 80 selected inpatient gender and mean age (standard deviation) were 33.8 and 66.3%, female and male, respectively, and 54 (±13.8) years old. The patients were submitted to different transplants, with kidney being the most prevalent type of surgery, representing 61.3%, followed by lung and liver in second and third place with 17.5 and 16.3% of frequency, respectively. All transplant types are listed in the Supplementary Table S1.

The patients included in the study were admitted to four of the seven hospitals from the complex. The number of patients admitted in each hospital was as follows: hospital A (HA) (61/80, 76.3%), hospital B (HB) (14/80, 17.5%), hospital C (HC) (1/80, 1.3%), and hospital D (HD) (4/80, 5.0%). Complete details of the hospital and units are in the Supplementary Table S1. The mortality rate of transplanted patients colonized or infected by CP-Kp was 21.3% (Supplementary Table S1).

### Bacterial Isolates

The 80 selected CP-Kp samples were collected from different sources, being 40% from surveillance rectal swabs and named as colonizing CP-Kp. Isolates obtained from clinical specimens were 21.2% from urine, 20.0% from the bloodstream, 16.2% from respiratory samples, 1.3% from abdominal liquid, and 1.3% from catheter tip (Supplementary Table S1). The carbapenemase genes detected were *bla*_KPC–__2_ (71 isolates, 88.8%), and *bla*_NDM–__1_ (nine isolates, 11.2%). The isolates were assigned to eight different STs: (1) ST11 with 62.5% of the isolates, (2) ST258 with 3.75%, (3) ST437 with 10%, these three STs were part of the clonal complex 11/258 (CC11/258); (4) ST15 with 8.75%, (5) ST4019 with 2.5%, both genetically related and the only carrying *bla*_NDM–__1_; (6) ST16 with 10%, (7) ST17 with 1.25%, both STs are part of the clonal complex 17 (CC17); and (8) ST39 with 1.25%. Isolates belonging to CC 11/258 are predominant in Brazil, as in the present study. On the other hand, other Brazilian studies described the presence of ST15 CP-Kp isolates, but to the best of our knowledge, isolates of these studies were not associated to *bla*_NDM–__1_, as described here (Gonçalves et al., 2017; Andrade et al., 2018). ST39 was not reported in previous studies with isolates obtained from patients in Brazil, and only individual cases of ST39 producing *bla*_KPC–__2_ or *bla*_NDM–__16_ were reported in two different studies from China (Liu et al., 2017; Xie et al., 2020).

Antibiotic resistance was observed against ampicillin, piperacillin-tazobactam, ampicillin-sulbactam, cefazolin, cefuroxime, cefepime, imipenem, meropenem, ertapenem, ciprofloxacin, norfloxacin, and nitrofurantoin to all CP-Kp isolates. On the other hand, 93.7% of the isolates were susceptible to amikacin, 18.7% to gentamicin, and 2.1% to sulfamethoxazole-trimethoprim. Susceptibility profiles of all clinical CP-Kp isolates are detailed in Supplementary Table S1. We recognize that the use of diffusion tests, instead of microdilution tests, can limit the definition of the resistance profile. However, agar diffusion tests are reliable and endorsed by both European Committee on Antimicrobial Susceptibility Testing and CLSI (Clinical and Laboratory Standards Institute, 2018; The European Committee on Antimicrobial Susceptibility Testing [EUCAST], 2018).

### Phylogenetic Analysis of KPC-Kp and NDM-Kp

**FIGURE 1:**
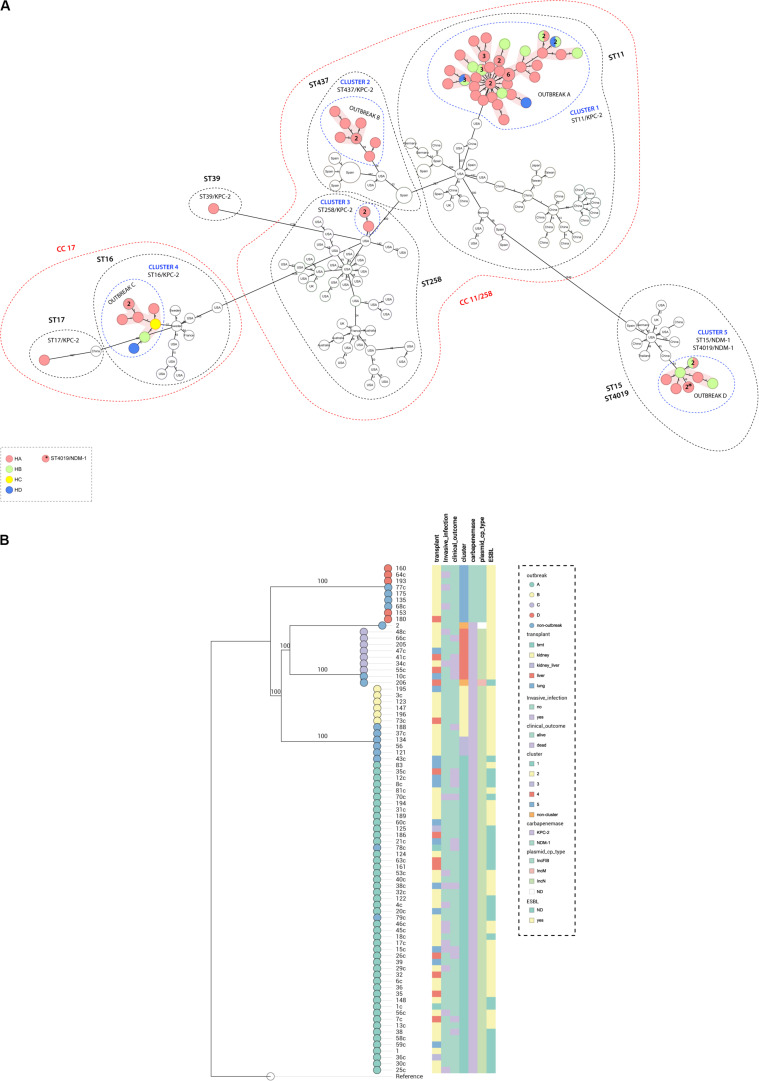
**(A)** Minimum spanning tree. Distance based on an *ad hoc cgMLST* of 2567 genes. Each colored circle indicates the hospital origin of the isolates; more than one isolate is indicated with its respective quantities in numbers, and non-colored circles are isolates from other countries. Red shadow indicates outbreak, blue dashed circles indicate cluster, black dashed circles represent sequence types, and red dashed circles represent clonal complex. Distances are not in scale. **(B)** Maximum likelihood tree showing the relationship between isolates, branch lengths are indicative of the number of SNPs. Colored strips in (from left to right) transplant type, invasive infection, clinical outcome, cluster, carbapenemase type, plasmid type carrying the *bla*_KPC–__2_ or *bla*_NDM–__1_ genes, and ESBL type. Nodes are colored according to the outbreak caused by the isolates. Reference: *K. pneumoniae* NTUH-K2044 (accession no. NC_012731.1).

**FIGURE 2:**
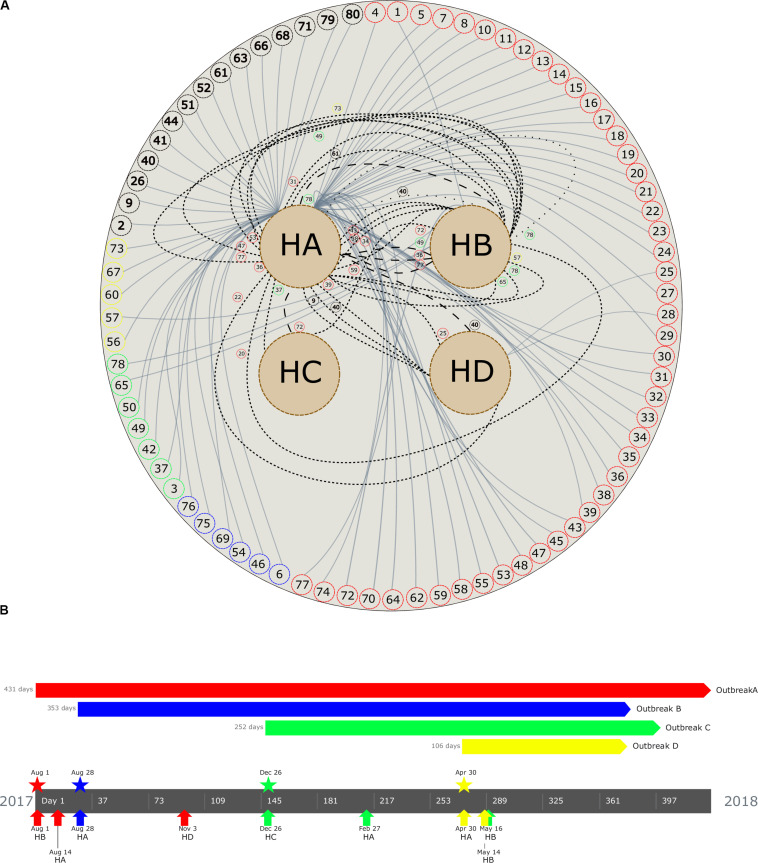
**(A)** Patient movement networks between hospitals. Outer dashed circles indicate patient’s identification being red (patients with isolates grouped in outbreak A), blue (outbreak B), green (outbreak C), yellow (outbreak D), and black (non–outbreak associated). Inner dashed circles indicate hospitals (HA, hospital A; HB, hospital B; HC, hospital C; and HD, hospital D). Continuous lines emerging in each patient represent hospitalization, short dashed lines represent first movement among hospital, long dashed lines represent second movement, and spaced dotted line represents third movement. All patient movements are identified with patient identification. **(B)** Timeline of the four carbapenemase-producing *K. pneumoniae* outbreaks. Outbreaks are represented by colors (outbreak A, red; B, blue; C, green; D, yellow). Pentagons indicates the period of the outbreak, stars represent the beginning of the outbreak, and arrows up indicate the first outbreak’s isolate detected in each hospital (see geolocation on https://microreact.org/project/RZLItlMKm).

Genome assemblies of the sequenced *K. pneumoniae* were analyzed by a gene-by-gene approach (Bialek-Davenet et al., 2014), together with all publicly available complete genomes of *K. pneumoniae* of the same STs; the allelic distance from cgMLST was visualized in a minimum spanning tree (Figure 1A). The results of allelic distances shown in Brazilian isolates had a population average difference of 836 (0–2,005) alleles and clustering into five groups (clusters 1–5). Four outbreaks (A–D) were detected when the following threshold was applied; less than 15 alleles distance in a pairwise comparison among all isolates (Lepuschitz et al., 2019; Miro et al., 2020). The isolates included in each outbreak presented an average difference of 12 (range = 0–25), 5 (range = 0–11), 4 (range = 0–11), and 2 (range = 0–5) alleles, in outbreak A (ST11/KPC-2), B (ST437/KPC-2), C (ST16/KPC-2), and D (ST15/NDM-1), respectively. Outbreak A was due to 47 isolates (colonizing 32%; clinical specimens 68%). They differed from outbreaks B, C, and D by an average of 355 (range = 346–363), 1,942 (range = 1,915–1,947), and 2,000 (range = 1,968–2,005) alleles. Outbreak B included six isolates (colonizing 66.7%; clinical specimens 33.3%); they differed from C and D by an average of 1,951 (range = 1,951–1,952) and 1.993 (range = 1,990–1,994). Outbreak C was due to seven isolates (colonizing 14.3%; clinical specimens 85.7%) that differed from outbreak D by an average of 1,991 (1,990–1,993) alleles. Outbreak D was due to five isolates (colonizing 80.0%, clinical specimens 20.0%). Both outbreaks A and B could maintain themselves during all the four collection quarters of sample collection. Outbreak A was present in three hospitals of the hospital complex (HA, HB, and HD) while outbreak B was present only in HA. Outbreak C was present in three hospitals (HA, HB, and HC) and started in the second period of sample collection and persisted until the end. Outbreak D was present in two hospitals (HA and HB) and started in the second half of the sample collection period (Figure 2B). Three outbreaks (A, C, and D) were present in at least two hospitals, suggesting that these *K. pneumoniae* high-risk clones (ST11/KPC-2, ST16/KPC-2, and ST15/NDM-1) were capable of interhospital spread and could be responsible for interregional and international spread, as reported elsewhere (Munoz-Price et al., 2013; Wu et al., 2019). We also compared CP-Kp isolated in this study with *K. pneumonia*e isolates of the same STs from other geographic regions; no isolate from another country was detected as a related clone with the Brazilian isolates; thus, we can affirm that we have the dissemination of geographically well-settled clones in our country (Figure 1A).

A maximum likelihood phylogenetic tree was reconstructed using 52,139 high-quality SNPs that were identified with reference to the sequence of *K*. *pneumoniae* strain NTUH-K2044 (Figure 1B). The general population presented SNP approximation average of 4,609 (0–11,982) SNPs and a clustering in five groups (clusters 1–5), similar to the clusters described previously with cgMLST analysis. The isolates were also grouped into four outbreaks (A–D). The isolates included in each outbreak presented an average difference of 16 (range = 0–31), 5 (range = 0–8), 7 (range = 1–17), and 1 (range = 0–3) SNPs, in outbreaks A (ST11/KPC-2), B (ST437/KPC-2), C (ST16/KPC-2), and D (ST15/NDM-1), respectively. Both techniques used to access the phylogeny of the CP-Kp isolates resulted in the same clusters and outbreaks. When a high-quality SNP approach was used, high diversity was found in isolates that belonged to ST11; this fact could be supported by the idea that some of these SNPs arose because of recombination events [30]. Patient movement networks between hospitals revealed that 45 of 47 patients involved in outbreak A were hospitalized at least once in the HA. Moreover, patient movement networks also revealed that after hospitalization patients could be moved once, twice, or even three times among the hospitals. Hospital A was a source hospital in 64.5% of the patient movements, thus being a significant reservoir to disseminate and acquire resistance features in CP-Kp (Figure 2A).

### Resistance and Virulence Genes in CP-Kp

**FIGURE 3:**
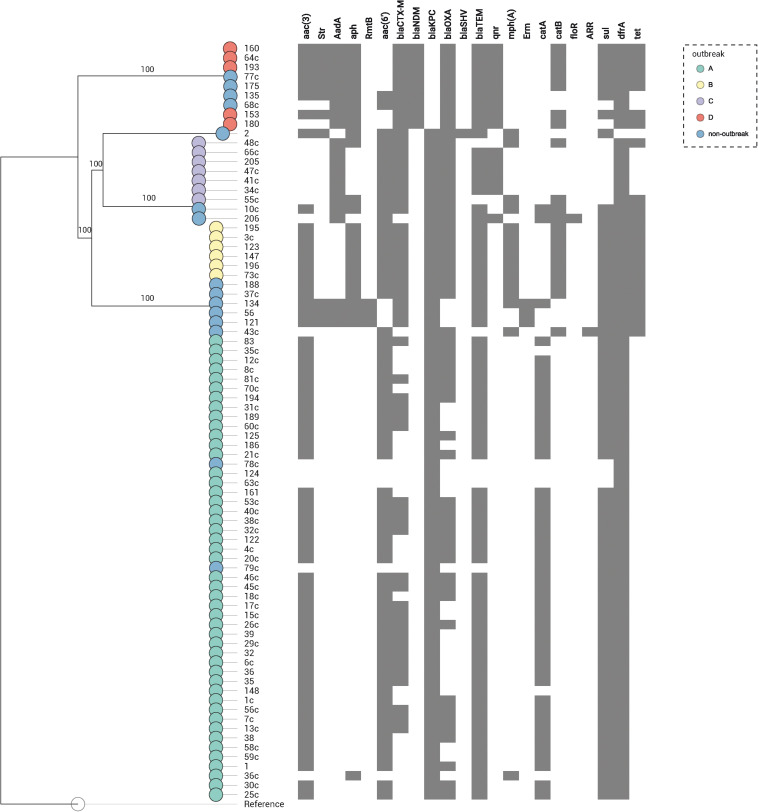
Distribution of the resistance genes in 80 *K*. *pneumoniae* expressing *bla*_KPC_ or *bla*_NDM_ genes. Genes conferring resistance (resistome) are shown at top. A map according to the presence or absence of each gene is shown underneath, where presence is shown in gray and absence in white. Nodes are colored according to the outbreak caused by the isolates. Reference: *K. pneumoniae* NTUH-K2044 (accession no. NC_012731.1).

In the 80 CP-Kp isolates studied, the average number of acquired resistance genes (ARGs) was 9 (range = 2–14, Supplementary Table S1 and Figure 3). Seventy-one (88.8%) carried *bla*_KPC–__2_, and nine (11.2%) carried *bla*_NDM–__1_. Extended spectrum β-lactamases genes (ESBL) identified in these isolates were as follows: 23 (28.7%) isolates carried *bla*_CTX–M–__2_, 20 (25%) *bla*_CTX–M–__15_, 5 (6.2%) *bla*_CTX–M–__8_, 4 (5%) *bla*_CTX–M–__14_, 1 (1.3%) *bla*_CTX–M–__35_, and 1 (1.3%) isolate carried *bla*_SHV–__40_. Coproduction with ESBL was detected in 62% of *bla*_KPC–__2_ *K. pneumoniae* and in 100% of *bla*_NDM–__1_ *K. pneumoniae* isolates. No plasmid-encoded AmpC genes were identified in our isolates. The predominant genes encoding aminoglycoside-modifying enzymes were N-acetyltransferases, being the most frequent *aac(3′)-IIa* (65%), *aac(6′)-Ib3* (55%), and *aac(6′)-Ib-cr* (17.5%). Three isolates (3.7%) carried acquired 16S rRNA methyltransferase *rmtB*; all these isolates were *bla*_KPC–__2_ producers and belonged to ST258. Plasmid-mediated quinolone resistance qnr-like determinants were detected in 18.8% of the isolates, being *qnrS1* the most frequent (11.3%). One study that performed WGS in 10 KPC-2-producing *K. pneumoniae* selected from different cities and states of Brazil found a high frequency of qnr-like resistant determinants (100%) and *bla*_CTX–M_ genes (80%) (Fuga et al., 2019). In contrast, we found only 8.4% and 62% of qnr-like determinants and *bla*_CTX–M_ genes, respectively. Chloramphenicol acetyltransferase *catA1* and *catB4* were the most frequent phenicol resistance mediators, being detected in 57.5 and 22.5% of the isolates, respectively. The dihydropteroate synthases associated with sulfonamide resistance also were detected; 67.5% presented *sul1* and 15.0% *sul2*; moreover, 2.5% of the isolates presented both *sul1* and *sul2* genes. The dihydrofolate reductases associated with trimethoprim resistance were detected in 98.8% of the isolates, being the most frequent *dfrA30* (77.5%). The isolates of outbreaks B and D presented a higher average number of ARGs when comparing with the isolates from the other outbreaks (outbreak B: average = 12, range = 12–12; outbreak D: average = 12, range = 10–13, outbreak C: average = 9, range = 8–10; outbreak A: average = 8, range = 2–9). To summarize, the most frequent ARG profile was as follows: *aac(3′)-IIa*, *aac(6′)-Ib3*, *bla*_CTX–M–__2_, *bla*_KPC–__2_, *bla*-_OXA–__2_, *bla*_TEM–__1__B_, *catA1*, *sul1*, and *dfrA30*, detected in isolates responsible by outbreak A (ST11/KPC-2). The most stable ARG profile was found in isolates from outbreak B (ST437/KPC-2), where all isolates carried the same profile: *aac(3′)-IId*, *aph(3′)-Ia*, *aac(6′)-Ib-cr*, *bla*_CTX–M–__15_, *bla*_KPC–__2_, *bla*-_OXA–__1_, *bla*_TEM–__1__B_, *mph(A)*, *catB4*, *sul1*, *dfrA30*, *tetA*, and *tetD* (Supplementary Table S1). The acquisition and loss of antibiotic resistance genes have been associated to *K. pneumoniae* strains undergoing selective antibiotic pressure (Simner et al., 2018). We noted that isolates within each outbreak did not have many changes in their ARG profile. The most relevant change was in isolates from outbreak A, which we observed an acquisition of *bla*_CTX–M_ genes in 51.1% of the isolates.

**FIGURE 4:**
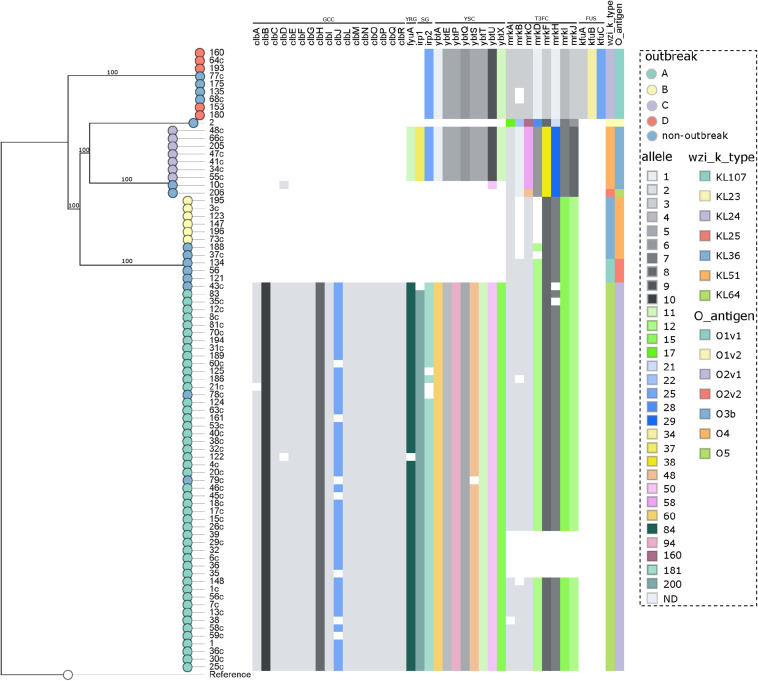
Distribution of the virulence genes in 80 *K. pneumoniae* expressing *bla*_KPC_ or *bla*_NDM_ genes. Genes conferring virulence (virulome) are shown at top. A colored heat map according to the allele number of each gene is shown underneath. Nodes are colored according to the outbreak caused by the isolates. Virulence genes groups are shown on top indicating (from left to right) genotoxin colibactin cluster (GCC), yersiniabactin receptor gene (YRG), siderophore genes (SG), yersiniabactin siderophore cluster (YSC), type III fimbriae cluster (T3FC), and ferric uptake system (FUS). Wzi alleles are shown in the top right. Reference: *K. pneumoniae* NTUH-K2044 (accession no. NC_012731.1).

The analysis of the capsular polysaccharide locus showed that all isolates included in each individual outbreak had the same *wzi* allele, not shared within outbreaks. The following K-loci were identified in outbreaks: KL64 (outbreak A), KL36 (B), KL51 (C), and KL24 (D), as shown in Supplementary Table S1 and Figure 4. Notably, KL1 to KL77 are associated with the classical 77 serologically defined K types. The most frequent K-locus detected in this study was the KL64, which was detected in the ST11 isolates. This K-locus is the most prevalent in China in ST11 isolates and has been reported in Brazilian ST11 *K*. *pneumoniae* isolates since 2018, which in one case was related to a fatal bacteremia, as we detected here in three patients (3.8%) which progressed to death (Andrade et al., 2018; de Campos et al., 2018). The intercontinental ST15 KL24 clone was found in seven isolates that coproduce *bla*_NDM–__1_ and *bla*_CTX–M–__15_; this capsular type is commonly linked to *bla*_CTXM–__15_ but not to *bla*_NDM–__1_ (Holt et al., 2015; Andrade et al., 2018).

The LPS O-antigen typing showed that, as in K-locus analyses, all isolates included in each individual outbreak had the same O-antigen allele, not shared within outbreaks. The O-antigens identified in the outbreaks were as follows: O2v1 (outbreak A), O4 (B), O3b (C), and O1v1 (D), as shown in Supplementary Table S1 and Figure 4. Its estimate that exists nine main O-antigens clusters described, and serotypes, O1, O2, and O3, are associated with almost 80% of the *K. pneumoniae* infections (Follador et al., 2016; Martin and Bachman, 2018). In contrast with the study reported by Wick et al. (2018), we observed a highly conserved O-antigen locus in the CC 11/258 isolates, being O2v1 the most frequent one, involved in the ST11 isolates.

The virulome was performed by WGS (Supplementary Table S1 and Figure 4), and in accordance to the BIGSdb-Kp database (Bialek-Davenet et al., 2014), a total of six virulence features were detected in this study, being the genotoxin colibactin cluster (*clbABCDEFGHIJLMNOPQR*), siderophore genes (*irp1* and *irp2*), the yersiniabactin siderophore cluster (*ybtAEPQSTUX*), the mannose-resistant *Klebsiella*-like (type III) fimbriae cluster (*mrkABCDFHIJ*), the ferric uptake system (*kfuABC*), and the yersiniabactin receptor gene *fyuA*. In general, the CP-Kp presented an average number of 27 (range = 6–36) virulence genes. A study recently conducted in São Paulo, Brazil, published that ST16 KPC-2 *K*. *pneumoniae* presented higher virulence than CC11/258 isolates in the *Galleria mellonella* pathogenicity model (Andrey et al., 2019). Although we had the limitation of not performing any pathogenicity animal model, in our study, the isolates of outbreak A (ST11/KPC-2) presented a higher average number of virulence genes when compared with the isolates from the other outbreaks (outbreak A: average = 35, range = 27–36; outbreak D: average = 20, range = 20–20; outbreak C: average = 19, range = 19–19; outbreak B: average = 6, range = 6–6), a fact that may enable ST11/KPC-2 isolates to be responsible for a high rate of invasive infections, as observed here and worldwide (Lee et al., 2016; de Campos et al., 2018). The colibactin cluster genes were only observed in outbreak A, being present in all the isolates of this group, but were disrupted in 17.0%. The colibactin-producing *K*. *pneumoniae* induce DNA damage and chromosomal instability in eukaryotic cells, which leads to senescence of epithelial cells and apoptosis of immune cells (Faïs et al., 2018), this virulence mechanism is strongly associated to CC23 *K*. *pneumoniae* (Lam et al., 2018).

The three virulence features included in yersiniabactin loci, *ybtAEPQSTUX*, siderophore genes, and yersiniabactin receptor, were detected in isolates of outbreaks A and C, and in all isolates of outbreak D, yersiniabactin loci lack yersiniabactin receptor and *irp1* gene, which could reduce the biosynthesis and expression of yersiniabactin, the adherence capability, and the pathogenicity of these isolates (Pelludat et al., 1998; Tu et al., 2016). The yersiniabactin cluster enables the scavenging of iron from host transport proteins, thus enhancing the ability of bacteria to replicate within the host and survive, and is mostly related to invasive infections (Holden and Bachman, 2015). The *ybtAEPQSTUX* cluster was detected in all outbreaks described in the study except only one that was not associated with an invasive infection (outbreak B). The type III fimbriae cluster (T3Fc) is responsible for mediating adherence to the renal tubular, respiratory tract, and lung tissue cells, which is crucial for biofilm formation (Schroll et al., 2010; Ares et al., 2017). Isolates from all outbreaks presented the T3Fc cluster, although in some isolates of outbreaks A and B, this cluster was not complete, and in a few isolates of outbreak A (12,8%, 6/47), T3Fc was not detected. The ferric uptake system (*kfuABC*) was detected only in outbreak D and non-outbreak related ST15 and ST4019 isolates. Interestingly, *bla*_NDM–__1_ carriers seem to be strongly associated with *kfuABC* system in our study. *KfuABC* has been associated with increased virulence by enabling lineages to use iron from diverse human and environmental sources, and it was recently associated to *K. pneumoniae* ST 101 (Roe et al., 2019).

To summarize, the most frequent virulence factor (VF) profile was as follows: *clb*ABCDEFGHIJLMNOPQR, *irp*1, *irp*2, *ybt*AEPQSTUX, *mrk*ABCDFHIJ, and *fyu*A, detected in isolates responsible by outbreak A (ST11/KPC-2). And the most stable VF profile was found in isolates from outbreak D (ST15/NDM-1), where all isolates carried the same profile: *irp*2, *ybt*AEPQSTUX, *mrk*ABCDFHIJ, *kfu*ABC (Supplementary Table S1).

We did not observe great differences when comparing isolates from rectal swabs or clinical specimens in terms of ST or outbreak. However, some differences could be observed as follows: (1) apparently, clinical specimens possessed less acquired resistance genes (ARGs) and more virulence factors (VFs) than surveillance isolates (Clinical specimens, ARGs: mean 8.5, range = 2–14, VFs: 29.9, range = 6–36; Rectal swabs. ARGs: 9.8, range = 2–14, VFs: 22.1, range = 6–36); (2) as expected, 88.2% (15/17) of the patients that died were infected by a CP-Kp, the remaining 2/17 (11.8%) were colonized but did not developed an infection; and (3) clinical specimens were collected from patients hospitalized in all four Hospitals, while the colonizing isolates were collected from patients hospitalized only in two Hospitals from the complex (Hospital A and B).

### Characterization of Plasmid Sequences Carrying *bla*_KPC_ and *bla*_NDM_ Genes

**FIGURE 5:**
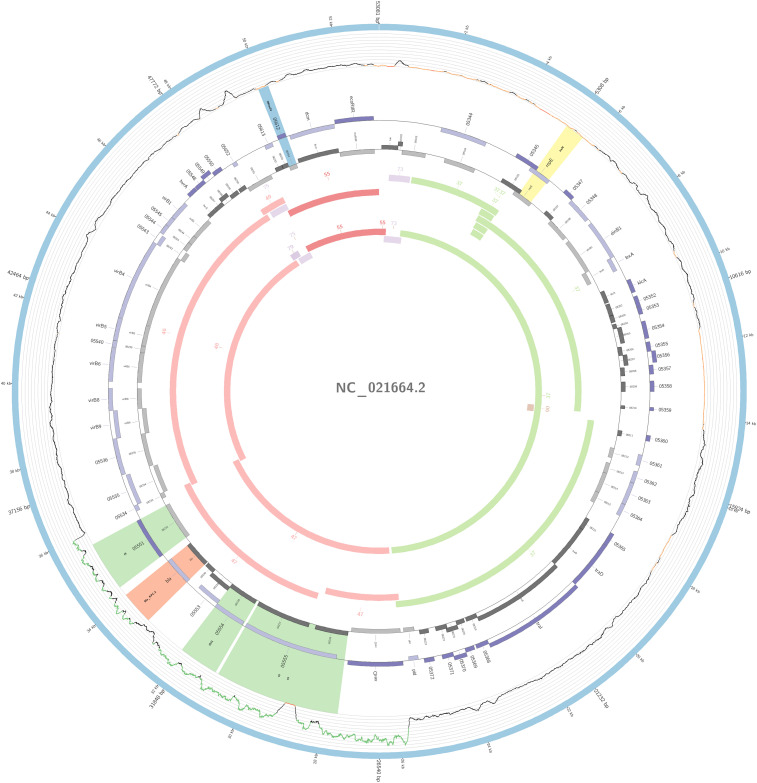
Overview of the IncN plasmid harboring *bla*_KPC–__2_ detected in *K. pneumoniae* ST11, ST16, ST258, and ST437 involved in outbreaks A, B, and C, in this study. The figure represents the *bla*_KPC_ plasmid according to the homology with highly similar one from database (blue outer ring). Graph represents the Illumina reads mapped against this plasmid with depth of coverage ranging from 0 (red) to 500, colored orange when values are 1 to 20, and green if more than 200 reads. Gray boxes represent cds from automatic annotation, with dark and light color when they were found in forward or reverse strand, respectively. Colored stripes represent a more detailed annotation that includes antibiotic resistance genes in red, IS in blue, and Rep genes in yellow. Homology between constructed plasmid and Illumina assembled contigs is represented in the inner ring, with each contig colored according to its number.

**FIGURE 6:**
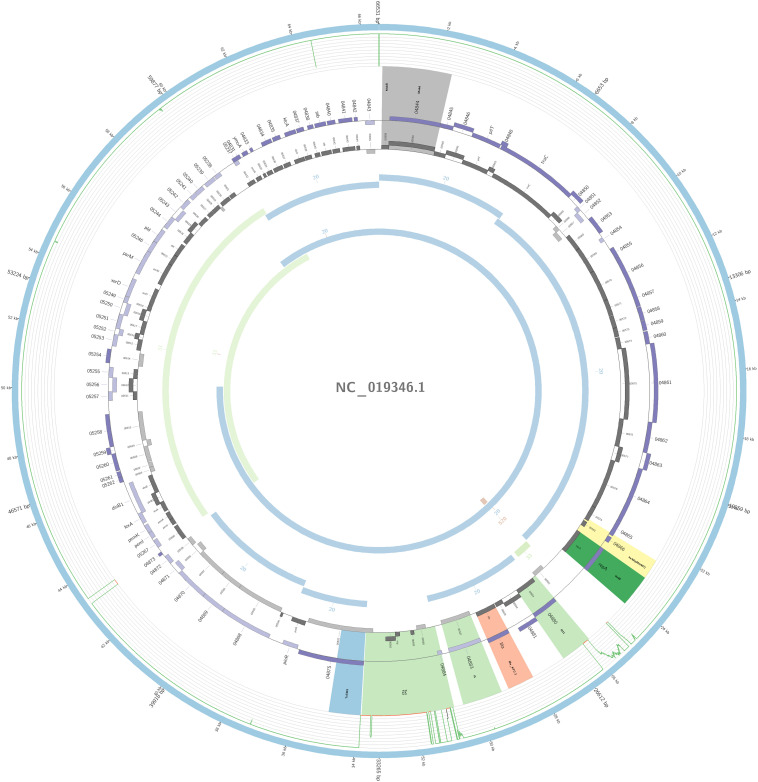
Overview of the IncM plasmid harboring *bla*_KPC–__2_ detected in *K. pneumoniae* ST17 non-outbreak involved in this study. The figure represents the *bla*_KPC_ plasmid according to the homology with highly similar one from database (blue outer ring). Graph represents the Illumina reads mapped against this plasmid with depth of coverage ranging from 0 (red) to 500, colored orange when values are 1 to 20, and green if more than 200 reads. Gray boxes represent cds from automatic annotation, with dark and light color when they were found in forward or reverse strand, respectively. Colored stripes represent a more detailed annotation that includes antibiotic resistance genes in red, IS in blue, and Rep genes in yellow. Homology between constructed plasmid and Illumina assembled contigs is represented in the inner ring, with each contig colored according to its number.

**FIGURE 7:**
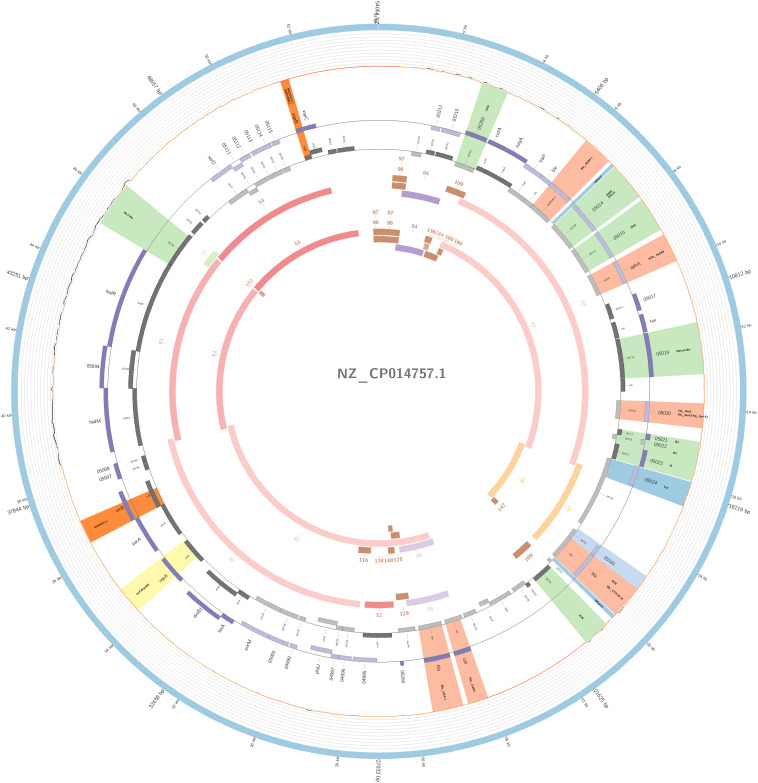
Overview of the IncFIB plasmid harboring *bla*_NDM–__1_ detected in *K. pneumoniae* ST15 and ST4019, involved in outbreak D in this study. The figure represents the *bla*_NDM_ plasmid according to the homology with highly similar one from database (blue outer ring). Graph represents the Illumina reads mapped against this plasmid with depth of coverage ranging from 0 (red) to 500, colored orange when values are 1 to 20, and green if more than 200 reads. Gray boxes represent cds from automatic annotation, with dark and light color when they were found in forward or reverse strand, respectively. Colored stripes represent a more detailed annotation that includes antibiotic resistance genes in red, IS in blue, and Rep genes in yellow. Homology between constructed plasmid and Illumina assembled contigs is represented in the inner ring, with each contig colored according to its number.

The plasmidID mapping tool was used for the identification and reconstruction of three plasmids harboring carbapenemase genes (two carrying *bla*_KPC–__2_ and one carrying *bla*_NDM–__1_). An IncN plasmid (∼53,081 bp), that was almost identical to the NC_021664.2 accession number (average identity = 95.68%; average coverage percentage = 94.57%), which carried a *bla*_KPC–2_ gene (Figure 5), an IncM plasmid (∼66,531 bp) highly similar to the plasmid NC019346.1 accession number (average identity = 98.04%; average coverage percentage = 95.92%), carrying a *bla*_KPC–2_ (Figure 6), and an IncFIB plasmid (∼54,064 bp) almost identical to the plasmid NZ_CP014757.1 accession number (average identity = 98.9%; average coverage percentage = 85.52%) that carried a *bla*_NDM–__1_ gene (Figure 7). In addition to the *bla*_NDM–__1_ gene, the plasmid IncFIB carried five ARGs (*bla*-_OXA–__1_, *catB4*, *bla*_CTX–M–__15_, *qnrS1*, and *aph(3′)-VI*), but no additional ARGs were identified in the IncN and IncM KPC-2 plasmids. Thus, the only plasmid that was associated with multidrug resistance (MDR) was the IncFIB, an IncF plasmid’s family known by their MDR characteristic (Rozwandowicz et al., 2018). The IncFIB plasmid was the only presenting the *bla*_NDM–__1_ in our study. IncFIB was also reported carrying the *bla*_NDM–__1_ in studies conducted in Myanmar and India, but differently from this study, and as had been described elsewhere, they found a greater number of plasmids harboring NDM-1 diversity (Hudson et al., 2014; Peirano et al., 2014; Conlan et al., 2016; Sugawara et al., 2018).

The IncN plasmid was detected in all isolates of outbreaks A, B, and C and in other non-outbreak related isolates belonging to ST11, ST16, ST258, and ST437; this fact highlighted the wide-spreading capacity of this plasmid among *K. pneumoniae* isolates. The genetic environment of this plasmid had a variant of Tn*4401b*, which carried only the *bla*_KPC–__2_ as ARG. The Tn*4401b* isoform also had the elements *tnpR*, *tnpA*, *istA*, and *istB* upstream of the *bla*_KPC–__2_ and downstream a truncated *tnpA* gene, highly similar to the already described FCF1305 plasmid (GenBank CP004366.2) (Perez-Chaparro et al., 2014). This finding confirms the persistence and movement of the IncN carrying *bla*_KPC–__2_ around the country.

In one ST17 non-outbreak causative isolate, *bla*_KPC–__2_ was harbored by an IncM plasmid. The IncM plasmid was often misidentified and reported as IncL/M group; this may explain why there are very few reports of the IncM harboring KPC-2 in Enterobacterales. The IncL/M group has been more associated with carrying the *bla*_OXA–__48_ gene (Kopotsa et al., 2019), but here in South America, it seems to play a role in the dissemination of the *bla*_KPC–__2_ gene; nevertheless, it was also reported in one study from China to be carrying the gene (Andrade et al., 2011; Liu et al., 2015; Jure et al., 2019). To our knowledge, ST17 *K. pneumoniae* with an IncM plasmid carrying the *bla*_KPC–__2_ has only been reported in this study. The isoform Tn*4401f* was detected in this plasmid carrying the *bla*_KPC–__2_ gene flanked upstream by the elements *istA* and *istB*, and downstream by *tnpA* and *tnpR*, a *bla*_KPC_ environment similar to the pNE1280 (GenBank JQ837276.1) (Bryant et al., 2013).

The IncFIB(pQil) plasmid was identified among outbreak D and in two additional non-outbreak related isolates belonging to ST15 and two to ST4019; all samples carried the *bla*_NDM–__1_ gene. Similar to pNDM-1fa (accession number NZ-CP014757.1) (Conlan et al., 2016), the IncFIB detected in the study collection has a region that contained a bleomycin resistance gene (*ble*_*MBL*_) upstream of the *bla*_NDM–__1_ gene, and downstream it had two transposases, IS*630* and IS*30*, and an aminoglycoside resistance gene (*aph(3′)-VI*). Furthermore, this plasmid also had another two resistance mediator genes, a fluoroquinolone (*qnrS1*) and an ESBL (*bla*_CTX–M–__15_).

## Conclusion

Our data reveal that high-risk clones in *K. pneumoniae* (ST11/KPC-2, ST16/KPC-2, and ST15/NDM-1) were responsible for the intrahospital and interhospital spread of *bla*_KPC–__2_ and *bla*_NDM–__1_ genes in transplanted patients from a hospital complex located in the south of Brazil. Patient movement networks among different hospitals may be associated with the dissemination of these clones.

We provided information about the phylogenetics of the study collection, showing a geographically conserved population in Brazil not clonally related to strains of the same MLST types isolated all over the world.

The IncN plasmid was the most frequent in our isolates and is probably a major cause of the widespread presence of *bla*_KPC–__2_ in this country. The dissemination of the *bla*_NDM–__1_ gene in the hospital complex studied was due to an IncFIB plasmid that harbored this carbapenemase gene together with the *bla*_CTX–M–__15_ gene.

Here we present a public health problem that needs to be more discussed and researched through further studies to better understand the spread, structural organization, and resistance/virulence features of the CP-Kp isolated in transplanted patients, in order to control and prevent outbreaks, given the great health and economic impact involved in these themes.

## Data Availability Statement

The datasets generated for this study can be found in the ENA project under accession number PRJEB34380.

## Ethics Statement

The studies involving human participants were reviewed and approved by the Research Ethics Committee from Santa Casa de Misericórdia de Porto Alegre and by the Plataforma Brazil of the Ministry of Health from Brazil. Number of registration and approval: 2.974.453; CAAE (Ethical appreciation presentation certificate): 67009717.7.0000.5335.

## Author Contributions

OR contributed to the conception, design, and implementation of the study, acquisition of laboratory and clinical data, analysis of the results, drafting the article, and approval of the final version of the manuscript. RS contributed to the acquisition of laboratory data, analysis of the results, and review and approval of the final version of the manuscript. EF contributed to the design of the study, acquisition of clinical data, analysis of the results, and review and approval of the final version of the manuscript. TS and CS contributed to the design of the study, analysis of the results, and review and approval of the final version of the manuscript. CD, JO, and MP-V contributed to the conception, design, and implementation of the study, analysis of the results, drafting the article, and approval of the final version of the manuscript. All authors contributed to the article and approved the submitted version.

## Conflict of Interest

The authors declare that the research was conducted in the absence of any commercial or financial relationships that could be construed as a potential conflict of interest.

## Footnotes

1 http://www.inkscape.org/

2 http://quast.sourceforge.net/quast

3 https://github.com/tseemann/snippy

4 http://bigsdb.pasteur.fr

5 https://github.com/katholt/Kaptive

6 https://github.com/BU-ISCIII/plasmidID
